# Supplementary material for: The Lysine Demethylases KdmA and KdmB Differently Regulate Asexual Development, Stress Response, and Virulence in Aspergillus fumigatus
Source: J Fungi (Basel). 2022 May 31;8(6):590. doi: 10.3390/jof8060590 (PMC9225160; doi:10.3390/jof8060590)
Supplement: Supplementary file 1 [file jof-08-00590-s001.zip › Table S1.pdf]

Table S1. Oligonucleotides used in this study.

Name	Sequence (5'→3')[a]	Purpose
oligo346	CCATGTGTGTCGAGTCCTTC	5' *ef1* for qRT-PCR normalization
oligo347	GAACGTACAGCAACAGTCTGG	3' *ef1* for qRT-PCR normalization
oligo303	GCTACCACTCTGCATCCTCA	5' *abaA* for qRT-PCR
oligo304	TACGAGCTCCAGCATGATTC	3' *abaA* for qRT-PCR
oligo305	ACGGCAGGAAGTTGTCTTCT'	5' *wetA* for qRT-PCR
oligo306	CTGTCAGCGACTTGTTGGAT	3' *wetA* for qRT-PCR
oligo256	TTCCAAGCAGAGCTTGTCAC	5' *brlA* for qRT-PCR
oligo257	CCAGGTTCTTTGCACTTGAA	3' *brlA* for qRT-PCR
oligo1557	TTCCACACCAACCTCGATGA	5' *imd2* for qRT-PCR
oligo1558	GAGACAAGGTTTCCGTCTGC	3' *imd2* for qRT-PCR
oligo1559	GGGTTGAAGGTGCAGGAGTA	5' *sdt1* for qRT-PCR
oligo1560	GTCTCCCTTTCCGCAAACTC	3' *sdt1* for qRT-PCR
oligo273	AGTCTCGGACGGACTGATCT	5' *laeA* for qRT-PCR
oligo247	GATACTTGTTGGCCACATCG	3' *laeA* for qRT-PCR
oligo271	AAATCCATCACATCCACCCT	5' *gliZ* for qRT-PCR
oligo272	GGTTGTTCATGGTCAGTTGC	3' *gliZ* for qRT-PCR

oligo1591	GCCCTATGCAGCTTTCTTCC	5' catalase for qRT-PCR
oligo1592	TCCATCATCCGCTCTCTTCC	3' catalase for qRT-PCR
oligo1593	GGTCAGTTCCAGCCTCTTCT	5' MnSOD for qRT-PCR
oligo1594	CTTGAAGTACTCGGCCTTGC	3' MnSOD for qRT-PCR
oligo1595	CTGGGAGAGAAGAAGCCCAA	5' thioredoxin reductase for qRT-PCR
oligo1596	GCATAGAAGAGACCGTTGGC	3' thioredoxin reductase for qRT-PCR
oligo1597	ACGCAGATCCTCAAGTCGAT	5' quinone oxidoreductase for qRT-PCR
oligo1598	GCCAGTGATCTCCTTCACCT	3' quinone oxidoreductase for qRT-PCR
oligo697	GCAATGTAAAGCTAACGTGCGTG	5' *AnpyrG* marker
oligo698	TGCCTTTAAGCTTCGGGTAGAG	3' *AnpyrG* marker
oligo1402	GCAGCCACCAAAGTTACCTT	5' flanking region of *AfukdmA*
oligo1403	*TTTGTAGGCTTTGGGCTGTTCA*CAATCGATAAAGATGAGAGCGCC	5' *AfukdmA* with *AnpyrG* tail
oligo1404	*CTGATCTACCCCTTGGAACGCAGCA*TGGAAGATGGTGCTTGCATG	3' *AfukdmA* with *AnpyrG* tail
oligo1405	TGAGCGCCAGTAAAGGAAGA	3' flanking region of *AfukdmA*
oligo1406	GGTGTTTCCCATCCTGCAAA	5' nested of *AfukdmA*
oligo1407	GATCGCAAGCGAAGAGTAAG	3' nested of *AfukdmA*
oligo1513	*TAGTTCTGTTACCGAGCCGG*CATGCAAGCACCATCTTCCA	3' *AfukdmA* with *hygR* tail for complementation
oligo1514	*GCTCTGAACGATATGCTCCAAC*ATGCTCTCTGTACATGCGTG	5' *AfukdmA* with *hygR* tail for complementation

oligo1396	TTACCTTGTCGTCCGTTTGC	5' flanking region of *AfukdmB*
oligo1397	*TTTGTAGGCTTTGGGCTGTTCA*CAAAGCTGGGTGGGGGACAAA	5' *AfukdmB* with *AnpyrG* tail
oligo1398	*CTGATCTACCCCTTGGAACGCAGCA*TTTCACGAACGGAAGCACTG	3' *AfukdmB* with *AnpyrG* tail
oligo1399	TTGGGTTGGATGCCGAAAAG	3' flanking region of *AfukdmB*
oligo1400	ATTCGACCCTTGCCTCATCA	5' nested of *AfukdmB*
oligo1401	GTTCGGCTTGATGATCGGTT	3' nested of *AfukdmB*
oligo1511	*TAGTTCTGTTACCGAGCCGG*CAGTGCTTCCGTTCGTGAAA	3' *AfukdmB* with *hygR* tail for complementation
oligo1512	*GCTCTGAACGATATGCTCCAAC*CCGATTGTATTCCTCTTCGG	5' *AfukdmB* with *hygR* tail for complementation
oligo1422	CCGGCTCGGTAACAGAACTANNNNNNNNNNGCCNNNNNNNNNN CAGAACGGCGTAACCAAAAGTCAC	5' of *hygR*
oligo1423	GTTGGAGCATATCGTTCAGAGCNNNNNNNNNNTAGNNNNNNNNN NTTCATCTTGACGACCGTTGATCTG	3' of *hygR*

[a] Tail sequence is in italic.
