# Supplementary figures and images for: The Lysine Demethylases KdmA and KdmB Differently Regulate Asexual Development, Stress Response, and Virulence in Aspergillus fumigatus
Source: J Fungi (Basel). 2022 May 31;8(6):590. doi: 10.3390/jof8060590 (PMC9225160; doi:10.3390/jof8060590)

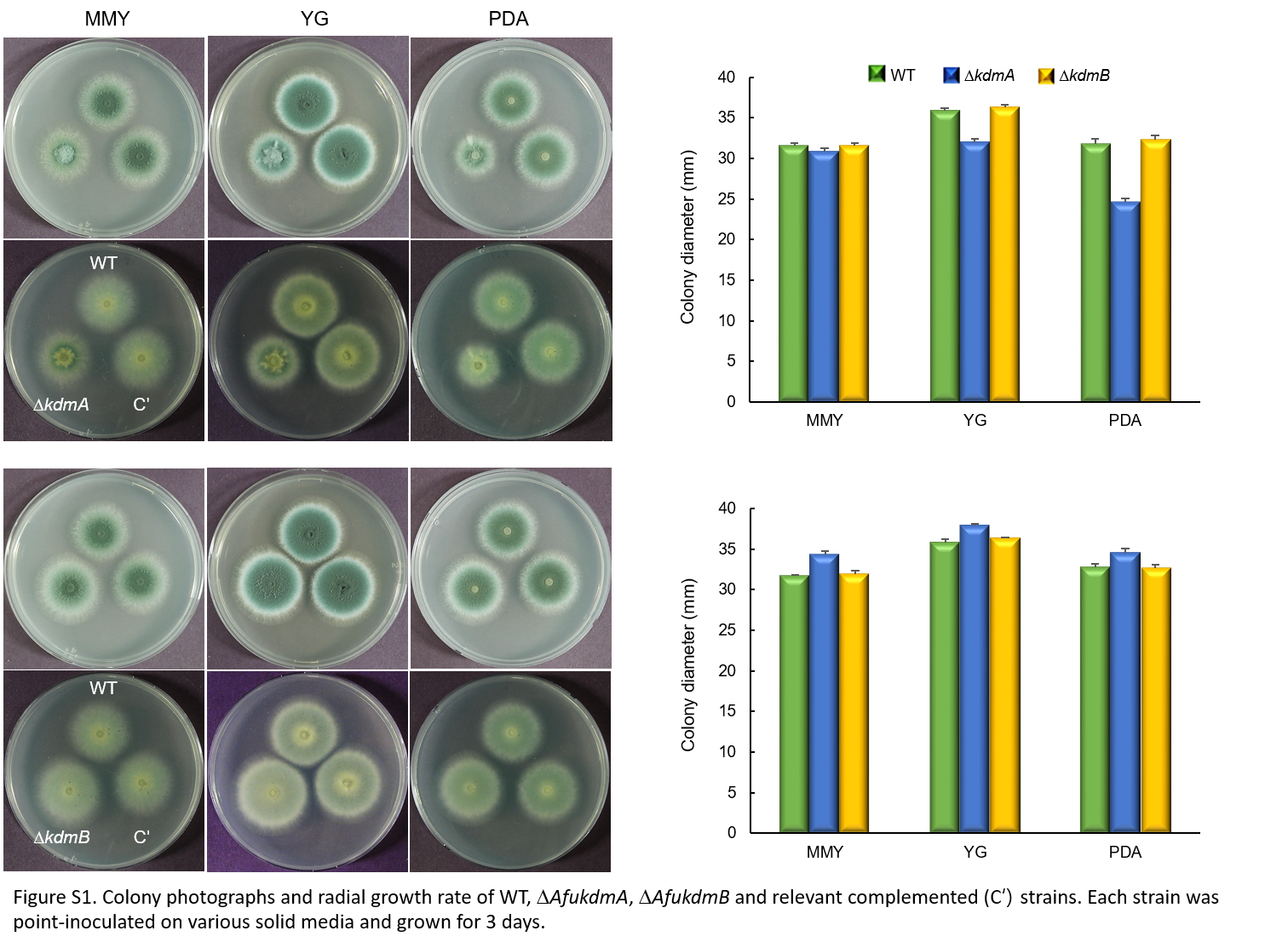

Supplement: Supplementary file 1 [file jof-08-00590-s001.zip › Fig. S1.tif]

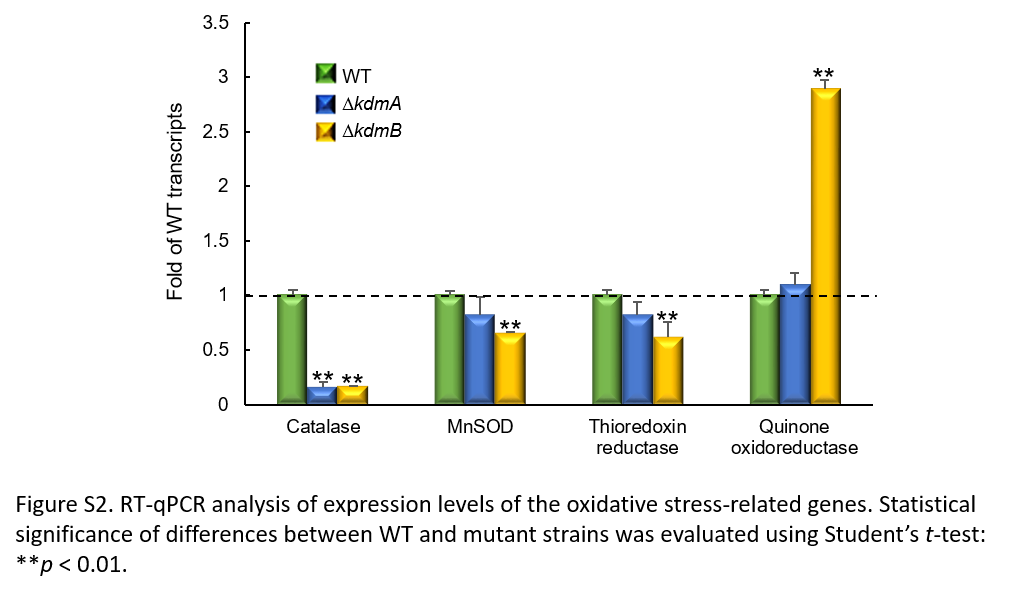

Supplement: Supplementary file 1 [file jof-08-00590-s001.zip › Fig. S2.tif]

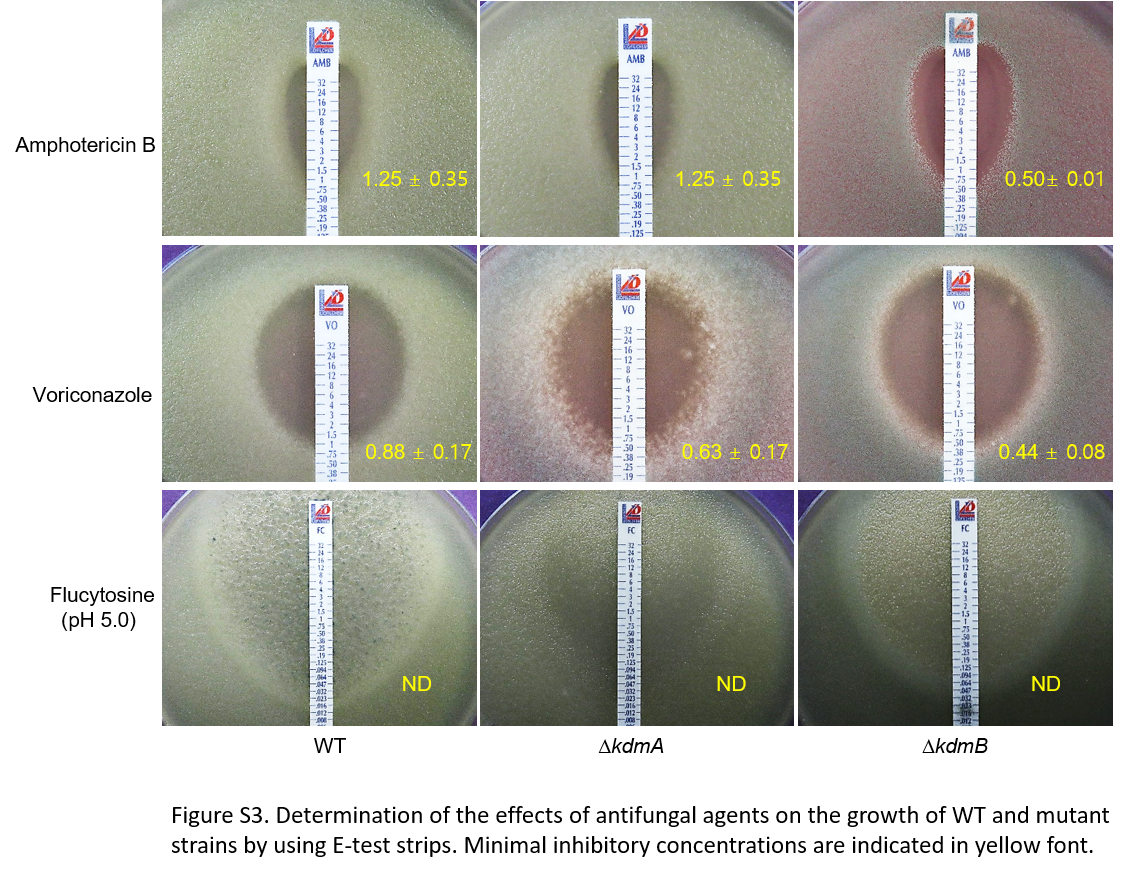

Supplement: Supplementary file 1 [file jof-08-00590-s001.zip › Fig. S3.tif]

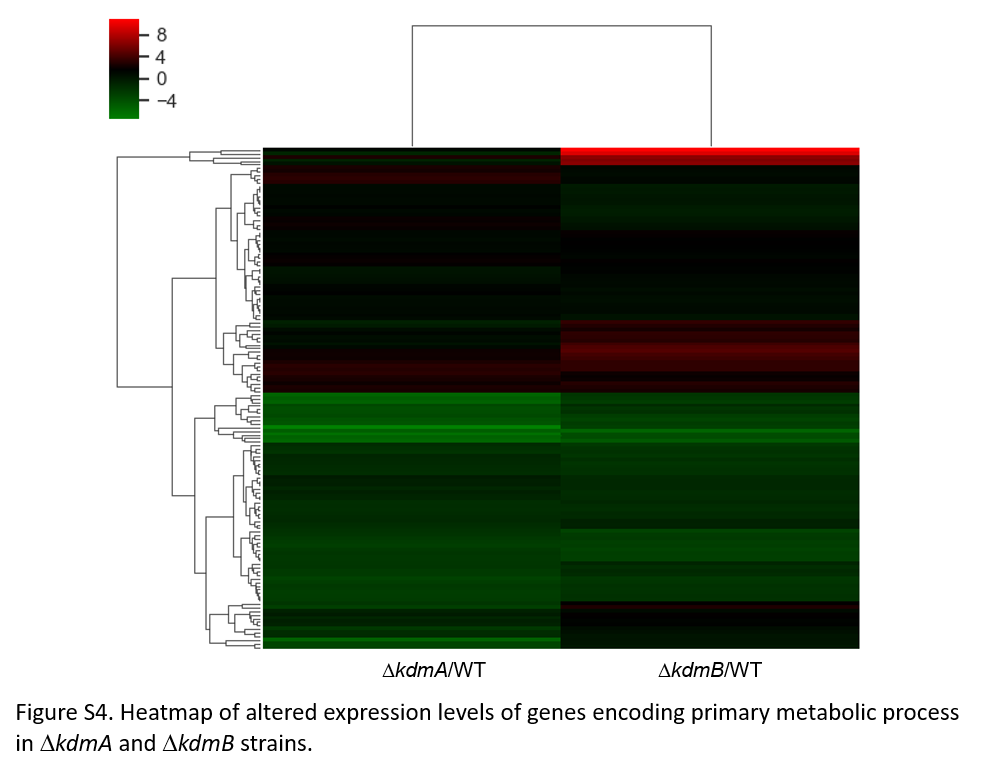

Supplement: Supplementary file 1 [file jof-08-00590-s001.zip › Fig. S4.tif]
